# Phosphorylation of the Chromatin Binding Domain of KSHV LANA

**DOI:** 10.1371/journal.ppat.1002972

**Published:** 2012-10-18

**Authors:** Crystal Woodard, Meir Shamay, Gangling Liao, Jian Zhu, Ai Na Ng, Renfeng Li, Rob Newman, Hee-Sool Rho, Jianfei Hu, Jun Wan, Jiang Qian, Heng Zhu, S. Diane Hayward

**Affiliations:** 1 High Throughput Biology Center, Johns Hopkins University School of Medicine, Baltimore, Maryland, United States of America; 2 Department of Pharmacology, Johns Hopkins University School of Medicine, Baltimore, Maryland, United States of America; 3 Department of Oncology, Johns Hopkins University School of Medicine, Baltimore, Maryland, United States of America; 4 School of Physiology & Pharmacology, University of Bristol, Bristol, United Kingdom; 5 Department of Opthalmology, Johns Hopkins University School of Medicine, Baltimore, Maryland, United States of America; University of North Carolina at Chapel Hill, United States of America

## Abstract

The Kaposi sarcoma associated herpesvirus (KSHV) latency associated nuclear antigen (LANA) is expressed in all KSHV associated malignancies and is essential for maintenance of KSHV genomes in infected cells. To identify kinases that are potentially capable of modifying LANA, in vitro phosphorylation assays were performed using an Epstein Barr virus plus LANA protein microarray and 268 human kinases purified in active form from yeast. Interestingly, of the Epstein-Barr virus proteins on the array, the EBNA1 protein had the most similar kinase profile to LANA. We focused on nuclear kinases and on the N-terminus of LANA (amino acids 1–329) that contains the LANA chromatin binding domain. Sixty-three nuclear kinases phosphorylated the LANA N-terminus. Twenty-four nuclear kinases phosphorylated a peptide covering the LANA chromatin binding domain (amino acids 3–21). Alanine mutations of serine 10 and threonine 14 abolish or severely diminish chromatin and histone binding by LANA. However, conversion of these residues to the phosphomimetic glutamic acid restored histone binding suggesting that phosphorylation of serine 10 and threonine 14 may modulate LANA function. Serine 10 and threonine 14 were validated as substrates of casein kinase 1, PIM1, GSK-3 and RSK3 kinases. Short-term treatment of transfected cells with inhibitors of these kinases found that only RSK inhibition reduced LANA interaction with endogenous histone H2B. Extended treatment of PEL cell cultures with RSK inhibitor caused a decrease in LANA protein levels associated with p21 induction and a loss of PEL cell viability. The data indicate that RSK phosphorylation affects both LANA accumulation and function.

## Author Summary

The Kaposi sarcoma associated herpesvirus (KSHV) is associated with cancers that have an increased incidence in individuals with compromised immune systems. KSHV expresses a protein, LANA, that is needed to maintain KSHV genomes in infected cells and also promotes the growth of KSHV associated tumors. Kinases regulate protein function through phosphorylation. To identify kinases that may affect LANA function, we performed a screen in which 268 human kinases were isolated and tested for the ability to phosphorylate LANA in vitro. We focused on the region of LANA that contains the chromatin binding domain, a motif essential for tethering KSHV genomes to the cell chromatin and maintaining latent infection. We identified serine 10 and threonine 14 as amino acids within the chromatin binding domain whose phosphorylation was important for histone binding. Serine 10 and threonine 14 were targets of the CK1, PIM1, GSK-3 and RSK3 kinases. Treatment with an inhibitor of RSK kinase reduced LANA binding to histones, decreased LANA protein levels and caused a loss of KSHV infected PEL cell viability. Our experiments show that phosphorylation affects LANA function and suggest that KSHV infected cells may be particularly vulnerable to kinase inhibitors.

## Introduction

The Kaposi sarcoma associated herpesvirus (KSHV) LANA protein is essential for establishment of KSHV latency through its role in replicating the KSHV genome, tethering the episomal genomes to cell chromosomes, interfering with induction of the viral lytic program and creating an environment that is permissive for cell survival and proliferation. Deletion of LANA in KSHV or rhesus rhadinovirus results in a more actively replicating virus [1], [2] and this outcome derives in part from loss of LANA mediated repression of the lytic RTA transactivator [3]–[6]. LANA promotes cell survival through induction of components of the Notch pathway [7], [8], by limiting p53 mediated cell death [9]–[11] and through inhibition of TGF-beta signaling [12]. LANA promotes cell growth by stabilizing beta catenin [13], deregulating c-Myc [14], [15], upregulating survivin and Id-1 expression [16], [17] and E2F transcriptional activity [18], [19] and modifying miRNA [20] and cell gene expression [21]. The effects on cell gene expression are due, in part, to LANA mediated de novo promoter methylation [22] and LANA interaction with a variety of transcription factors [14], [15], [23]–[31].

LANA serves as the origin binding protein for KSHV latency DNA replication and binds to sequences within the terminal repeats [32]–[34] to support latent DNA replication [35]–[37] and episomal DNA persistence [38], [39]. LANA appears as nuclear speckles in KSHV infected cell nuclei. This speckling pattern requires the presence of KSHV DNA and in the absence of viral genomes LANA displays a nuclear diffuse staining pattern. LANA links KSHV episomes to host cell chromosomes and maintenance of the KSHV episomes in replicating cells is dependent on this LANA interaction [40]. LANA interaction with histones H2A and H2B through the N-terminal chromatin binding domain is critical for LANA association with chromosomes [41], [42]. However, both N-terminal and C-terminal regions of LANA bind to chromatin [43]–[45] and LANA also interacts with other chromosome associated proteins such as MeCP2, Brd4, DEK, HP-1 alpha and CENP-F [18], [44], [46]–[50].

The LANA primary amino acid sequence includes 120 serine, threonine and tyrosine residues that could be subject to post-translational modification. The kinases glycogen synthase kinase 3, PIM1/3, ERK1/2 and DNA-PK, [51]–[55] have been shown to phosphorylate LANA and RSK1 has been shown to interact with LANA [55]. However, there has been no global analysis of kinases that are potentially capable of modifying LANA function through phosphorylation. We screened 268 human kinases for the ability to phosphorylate LANA in vitro using a protein array format that also included Epstein-Barr virus proteins. The presence of serine and threonine residues in the N-terminal LANA chromatin binding domain led us to a focus on this motif. The assays validated CSK1, PIM1, GSK-3 and RSK3 as kinases that phosphorylated the critical serine 10 and threonine 14 residues in the chromatin binding domain of LANA and RSK as a kinase family whose inhibition affected LANA interaction with histone H2B, LANA protein levels and PEL cell viability.

## Results

### Screening for human kinases that phosphorylate LANA

**Figure 1:**
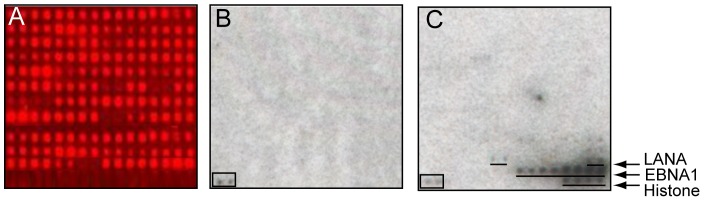
Phosphorylation assays on the EBV plus LANA protein array. A. Protein array probed with anti-GST antibody followed by CY5-labeled secondary antibody. Note that the EBNA1 (392–641)-V5-6xHis, 6xHis-Biotin AviTag-LANA (1–329) and control proteins are not expressed as GST-fusions and are not detected with anti-GST antibody. B. Control incubation with [γ^32^P]-ATP in kinase buffer. C. Incubation with [γ^32^P]-ATP, kinase buffer plus casein kinase 1, gamma 2 (CSNK1G2). Boxed signal, NME1 kinase control.

**Figure 2:**
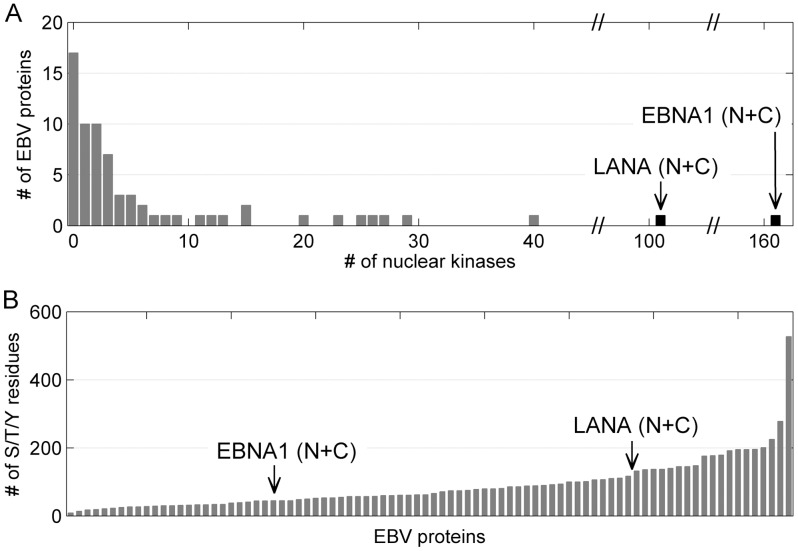
Relative phosphorylation of KSHV LANA and EBV EBNA1. A. Histogram comparing the number of human kinases phosphorylating individual EBV proteins on the protein array with the number recognizing KSHV LANA and EBV EBNA1. B. Histogram comparing the number of serine (S), threonine (T) and tyrosine (Y) residues in individual EBV proteins with the number in KSHV LANA and EBV EBNA1.

We have previously described a protein microarray displaying Epstein–Barr virus (EBV) proteins purified as GST-fusions from yeast and printed in duplicate [56]. This array was additionally printed with a series of EBV EBNA1 and KSHV LANA N-terminal and C-terminal polypetides printed either as 6xHis-GST fusions, V5-6xHis fusions or 6xHis-Biotin AviTag fusions. The array also contained control proteins that were used for orientation and normalization. This platform was used to globally identify human kinases that phosphorylate the KSHV LANA protein. Two hundred and sixty-eight human kinases were purified from yeast in active form as assayed by dot blot phosphorylation assays using a mixture of histone H3, myelin basic protein and casein as the substrate. Phosphorylation assays were performed using kinase buffer containing [γ^32^P]-ATP and individual kinases. As a negative control, two chips per experiment were incubated with kinase buffer containing [γ^32^P]-ATP but minus the protein kinase. Phosphorylation signals were detected by exposing the arrays to X-ray film. An example of a kinase assay performed on the protein array is shown in Figure 1. In the subsequent analyses performed with GenePix software, paired signals that were 3 standard deviations (SD) above background were considered positive. These assays identified 101 known or predicted nuclear kinases that phosphorylated KSHV LANA (N+C). The EBV EBNA1 protein is the functional homolog of KSHV LANA although the two proteins have no significant amino acid homology. Interestingly, 99 of the kinases that phosphorylated KSHV LANA also phosphorylated EBV EBNA1, a striking degree of overlap (Table S1). KSHV LANA and EBV EBNA1 were also phosphorylated by significantly more kinases than any of the other EBV proteins on the array (Figure 2A). The median number of nuclear kinases phosphorylating the other EBV proteins on the array was 2. The extensive phosphorylation of EBV EBNA1 and KSHV LANA was not due to a disproportionate representation of serine, threonine and tyrosine residues in these proteins relative to the other EBV encoded proteins (Figure 2B). EBNA1 and LANA proteins contain 45 and 120 serine, threonine and tyrosine residues respectively. The median number for EBV encoded proteins is 62.

### Phosphomimetic mutations of S10 and T14 restore LANA binding to histone H2B

**Figure 3:**
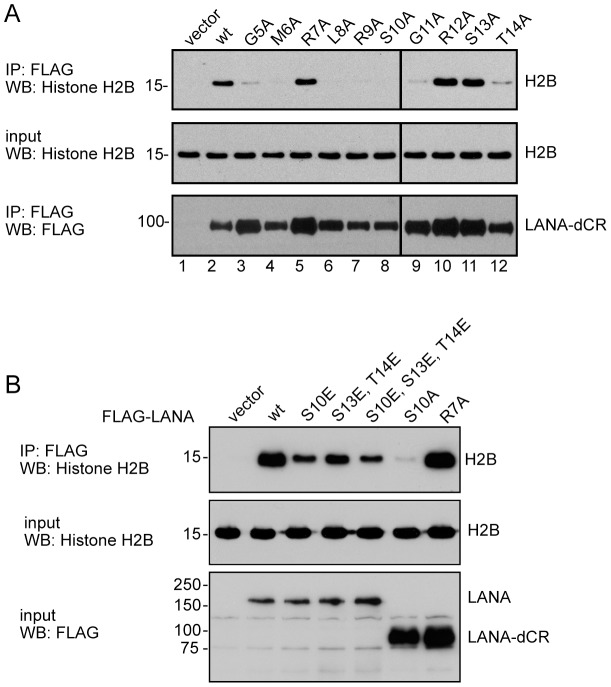
Phosphomimetic mutations of LANA S10 and T14 rescue histone binding. A. Western blots showing the effect on binding to histone H2B of individual alanine substitutions across the LANA chromatin binding domain. Flag-LANA was immunoprecipitated from transfected HEK293T cells and the bound endogenous H2B was detected using anti-H2B antibody. B. Western blots showing the effect of individual and grouped phosphomimetic mutations at S10, S13 and T14 on LANA binding to H2B. Flag-LANA or Flag-LANA deleted for the central repeats (dCR) was immunoprecipitated from transfected HEK293T cells and the bound endogenous H2B was detected using anti-H2B antibody.

Amino acids 5–22 are important for KSHV association with the cell chromatin [41], [42], [57]. Within this domain are three serine residues and two threonine residues that could be modified by phosphorylation. Previous mutagenesis studies found that mutation of S10 to alanine individually or in the context of a triple mutation prevented chromatin binding, as did mutation of S13 to alanine in the context of a triple mutation. Mutation of T14 to alanine was +/− for chromatin binding, mutation of S22 did not affect binding and mutation of T19 was not examined [41], [57]. Chromatin binding correlated with binding to histones H2A and H2B [41]. The S13A mutation has been examined only in the context of a triple alanine mutation. We consequently first examined the effect on binding to endogenous histone H2B of individual alanine mutations of LANA residues 5 through 14, including S13. In immunoprecipitation assays performed on transfected HEK 293T cells the observed loss of binding of Flag-LANA G5A, M6A, L8A, R9A, S10A, G11A, the retention of binding by R7A and the weak +/− binding of T14A recapitulated published results (Figure 3A). As an individual mutation, conversion of serine 13 to alanine did not affect binding to histone H2B. The mutagenesis data implies that, if phosphorylation impacts on LANA's ability to bind histones, the relevant residues would be S10 and T14.

To assess the potential contribution of phosphorylation of the chromatin binding domain, LANA variants were generated that carried phosphomimetic mutations of S10, S13 and T14. The ability of these LANA variants to immunoprecipitate histone H2B was examined. Conversion of S10 to glutamic acid (S10E) restored the ability to bind to H2B both in the context of an individual S10E mutation and in the context of an S10E, S13E, T14E triple mutation (Figure 3B). LANA carrying the T14E, S13E double mutation also showed increased histone H2B binding over that seen with T14A. Since mutation at S13 to a non-phosphorylatable alanine residue did not affect binding, this change can be attributed to T14E. These observations suggest that phosphorylation of S10 and T14 may impact on LANA function.

### Kinases that phosphorylate the N-terminal 50 amino acids of LANA

**Figure 4:**
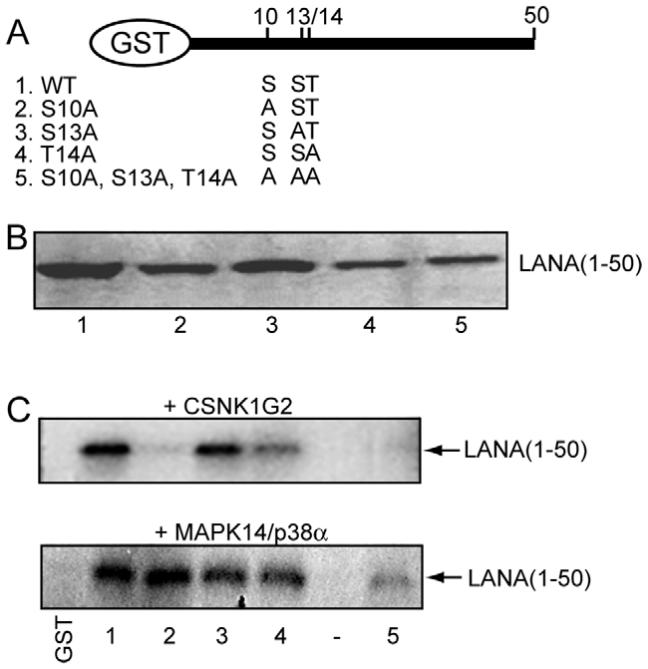
Phosphorylation of GST-LANA(1–50). A. Diagram of the GST-LANA aa 1–50 wild-type (1) and mutant constructions (2–5) showing the amino acid changes at positions 10, 13 and 14 in the chromatin binding domain. B. Coomassie brilliant blue staining of the purified GST-LANA(1–50) wild-type (1) and mutant (2–5) proteins used in the phosphorylation assays. C. Examples of *in vitro* phosphorylation assays in which GST-LANA(1–50) wild-type and mutant proteins were incubated with CSNK1G2 or MAPK14/p38a kinases. (−), minus peptide.

The LANA N-terminus contains the chromatin binding domain and we therefore chose to focus on this region of LANA and to limit subsequent experiments to the 63 kinases that phosphorylated both the 6xHis-GST and 6xHis-Biotin AviTag fusions of the N-terminus of LANA (aa 1–329) and are known or predicted to function in the cell nucleus. LANA has a nuclear localization and this subset of kinases is therefore more likely to be biologically relevant (Table 1). Included in this list are the PIM1 and GSK-3 kinases that are known to phosphorylate LANA [51], [53], [54]. We generated GST-fusion proteins of the N-terminal 50 amino acids of LANA along with derivatives in which serine or threonine residues within the chromatin binding domain (S10, S13, T14) were mutated to alanine individually or as a triple mutation (Figure 4A). The purified GST-fusion proteins used in the kinase assays are shown in Figure 4B. In vitro kinase assays were performed with 42 of the kinases that phosphorylated LANA(1–329) on the protein array and 31 of these kinases phosphorylated the N-terminal 50 amino acids of LANA (Table 1). Examples of the kinase assay using these GST-LANA (1–50) proteins as substrates are shown in Figure 4C. MAPK14/p38α (mitogen activated protein kinase 14) phosphorylated GST-LANA(1–50), each of the S10A, S13A and T14A mutants and the S10A, S13A, T14A triple mutant. MAPK14 either phosphorylates LANA 1–50 outside of the chromatin binding domain or alternatively, if S10, S13 or T14 are sites, then MAPK4 must also be capable of phosphorylating the N-terminus outside of this domain. CSNK1G2 (casein kinase 1, gamma 2 isoform) phosphorylated GST-LANA(1–50) and the S13A and T14A mutants but not the S10A or S10A,S13A,T14A mutants. This suggests that S10 within the chromatin binding domain is phosphorylated by casein kinase 1.

**Table 1:** Nuclear kinases that phosphorylate N-terminal regions of LANA.

Gene Name	1–340	1–50	3–21	Gene Name	1–340	1–50	3–21
CAMK1D	+	−	−	MARK2	+	+	+
CAMK4	+	+	−	MAST1	+	+	+
CAMKV	+	−	−	MET	+	NT	NT
CDK1	+	−	−	MKNK2	+	NT	NT
CDK5	+	−	−	MYO3A*	+	NT	NT
CDKL5	+	−	−	NEK3	+	−	−
CHEK2	+	−	−	NEK10*	+	+	+
CSNK1A1L	+	NT	NT	NPR2	+	NT	NT
CSNK1D	+	−	−	NUAK2	+	+	−
CSNK1G2*	+	+	+	PAK4*	+	NT	NT
CSNK2A1	+	−	−	PAK6	+	+	+
DMPK	+	NT	NT	PDPK1*	+	NT	NT
DYRK1B	+	+	+	PIM1	+	+	+
FASTK	+	−	−	PLK4	+	+	+
FGFR1	+	NT	NT	PRKACB	+	NT	NT
FGFR4	+	NT	NT	PRKCD	+	+	+
GRK5	+	+	+	PRKCZ	+	NT	NT
GSK3β	+	+	+	PRKX	+	+	+
HIPK1	+	+	+	PTK2	+	NT	NT
HIPK4	+	NT	NT	PTK6	+	+	+
IKBKB	+	NT	NT	RPS6KA2	+	+	+
IRAK1	+	+	+	RPS6KA4	+	+	+
IRAK3	+	NT	NT	RPS6KA5	+	+	+
KIT	+	+	−	RPS6KB1	+	−	−
MAK	+	+	+	SCYL2	+	NT	NT
MAP2K1*	+	+	+	SGK2	+	NT	NT
MAP2K3	+	+	−	STK31*	+	+	+
MAP2K7	+	+	+	STK40	+	+	−
MAP3K7	+	+	+	TTK*	+	NT	NT
MAPK13	+	NT	NT	WEE1	+	+	−
MAPK14	+	+	+	WNK1*	+	NT	NT
MAPK8	+	+	−				

* predicted nuclear kinases.

NT, not tested.

### Kinases that phosphorylate LANA amino acids 3–21

**Figure 5:**
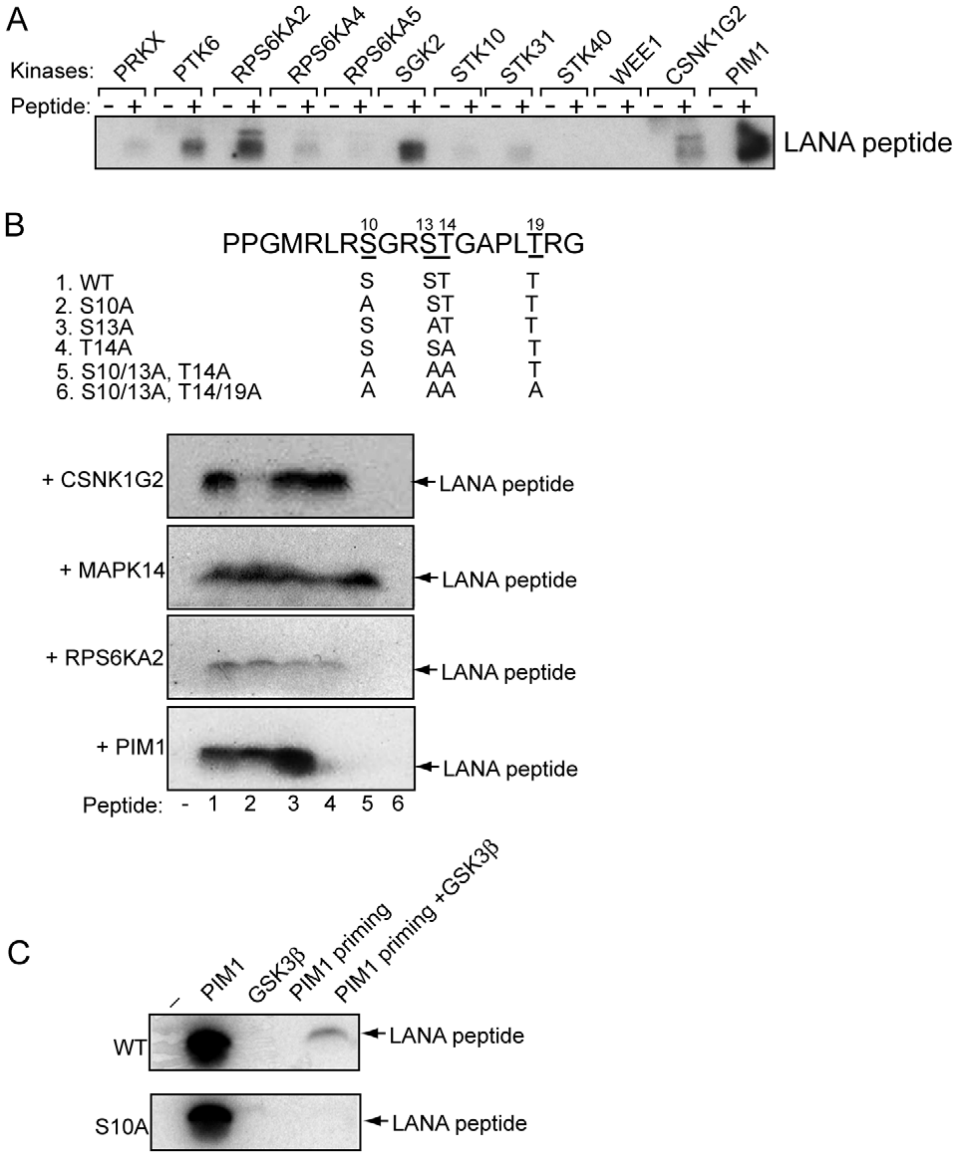
Identification of kinases that phosphorylate the LANA chromatin binding domain. A. Phosphorylation of a LANA aa3–21 peptide by the indicated kinases. The peptide was separated from the kinase reaction mixture by electrophoresis and the gel dried and subjected to autoradiography. B. Upper: Amino acid sequence of the wt LANA 3–21 peptide (1) showing the positions of the single and grouped sequence changes in the mutant peptides (2–6). Lower: Examples of phosphorylation of the wild-type and mutant peptides by the indicated kinases. C. GSK-3 phosphorylates S10 after PIM1 priming. Wild-type and S10A mutant peptides were incubated with PIM1 or GSK-3β or with PIM1 plus unlabelled γ-ATP (PIM1 priming) or with GSK-3 β after PIM1 priming followed by inhibition of PIM1 with the PIM1 inhibitor SMI-4a. (−), minus kinase.

The possibility that phosphorylation of sites outside of the chromatin binding domain could complicate interpretation of the kinase assays using GST-LANA (1–50) as substrate led us to turn to peptide substrates that covered only the LANA chromatin binding domain. A synthetic peptide representing LANA amino acids 3–21 and covering the S10 and T14 amino acids of interest was used to retest 31 of the nuclear kinases that were positive for phosphorylation of GST-LANA (1–50). In this assay, 24 of these kinases also phosphorylated the amino acid 3–21 LANA peptide (Table 1). Examples of the peptide phosphorylation assay are shown in Figure 5A.

We next performed in vitro phosphorylation assays using synthetic peptides that carried individual mutations in S10A, S13A or T14A or combined mutations of S10A,S13A,T14A or S10A,S13A,T14A,T19A (Figure 5B). In this set of assays we identified CSNK1G2, PIM1 and RSK3 as kinases that phosphorylated S10 or T14 within the chromatin binding domain. CSNK1G2 phosphorylated the wild-type peptide and S13A and T14A mutant peptides but not the S10A or S10A,S13A,T14A or S10A,S13A,T14A,T19A peptides (Figure 5B). This is consistent with the data obtained using GST-LANA(1–50) which also implicated casein kinase 1 in phosphorylation of S10. PIM1 phosphorylated the wild-type, S10A and S13A peptides but not the T14, S10A,S13A,T14A or S10A,S13A,T14A,T19A peptides indicating that T14 is a site for PIM1 phosphorylation (Figure 5B). This observation differs from a previous study in which PIM1 phosphorylation of GST-LANA was linked specifically to S205 and S206 [51]. RSK3 (RPS6KA2) phosphorylated the wild-type, S10A, S13A and T14A peptides but not the S10,S13,T14 or S10A,S13A,T14A,T19A peptides (Figure 5B). This is consistent with RSK3 phosphorylation of two or more of the S10, S13 and T14 residues. MAPK14/p38alpha phosphorylated all the peptides except the S10A,S13A,T14A,T19A peptide indicating MAPK14 phosphorylation at T19 (Figure 5B).

GSK-3 usually requires a priming phosphorylation by another kinase at the +4 position. The activity of GSK-3 on the LANA proteins purified from yeast implies that some of the protein was being purified in a pre-primed state. However, the synthetic peptide substrate was not phosphorylated by GSK-3 (Figure 5C). Pre-incubation of the peptide with PIM1 in the presence of unlabelled γ-ATP followed by incubation with GSK-3β in the presence of [γ^32^P]-ATP and a PIM1 inhibitor, SMI-4a, resulted in phosphorylation of the wild-type peptide but not of the S10 mutant peptide (Figure 5C). Thus PIM1 phosphorylation at T14 is able to prime for GSK-3β phosphorylation at S10.

### Kinase inhibition affects histone H2B interaction and LANA protein levels

**Figure 6:**
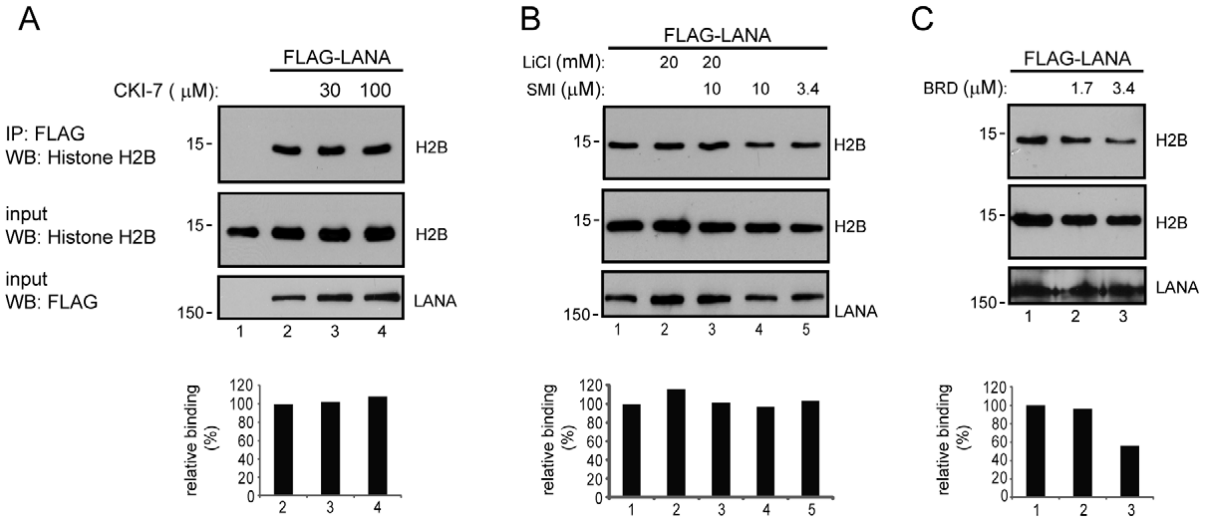
Impairment of LANA binding to H2B occurs upon short-term treatment with RSK inhibitor but not inhibitors of CKI, PIM1 or GSK-3. A. Cells treated with CKI inhibitor (CKI-7). B. Cells treated with GSK-3 inhibitor (LiCl) or PIM1 inhibitor (SMI-4a) individually or in combination. C. Cells treated with RSK inhibitor (BRD 7389). Western blots examine interaction between transfected Flag-LANA and endogenous histone H2B. Quantitation of the band densities provides a measure of relative binding (LANA bound H2B/input H2B) and was obtained using Image J. The ratio in untreated cells was set at 100%.

To examine the effect of kinase inhibition on LANA interaction with histone H2B, 293 cells were transfected with Flag-LANA and treated with the CK1 inhibitor CKI-7, the PIM1 inhibitor SMI-4a, the GSK-3 inhibitor LiCl, the RSK inhibitor BRD 7389 or with DMSO carrier. Short-term (4 hr) treatment with CKI-7 at 30 and 100 µM had no effect on LANA interaction with histone H2B (Figure 6A). Inhibition of GSK-3 or PIM1, individually or in combination, by treatment for 6 hr with LiCl and SMI-4a also had no impact on LANA binding to H2B (Figure 6B). However, treatment with 1.7 or 3.4 µM BRD 7389 for 6 hr decreased LANA interaction with histones in a dose responsive manner while having little effect on LANA protein levels (Figure 6C).

**Figure 7:**
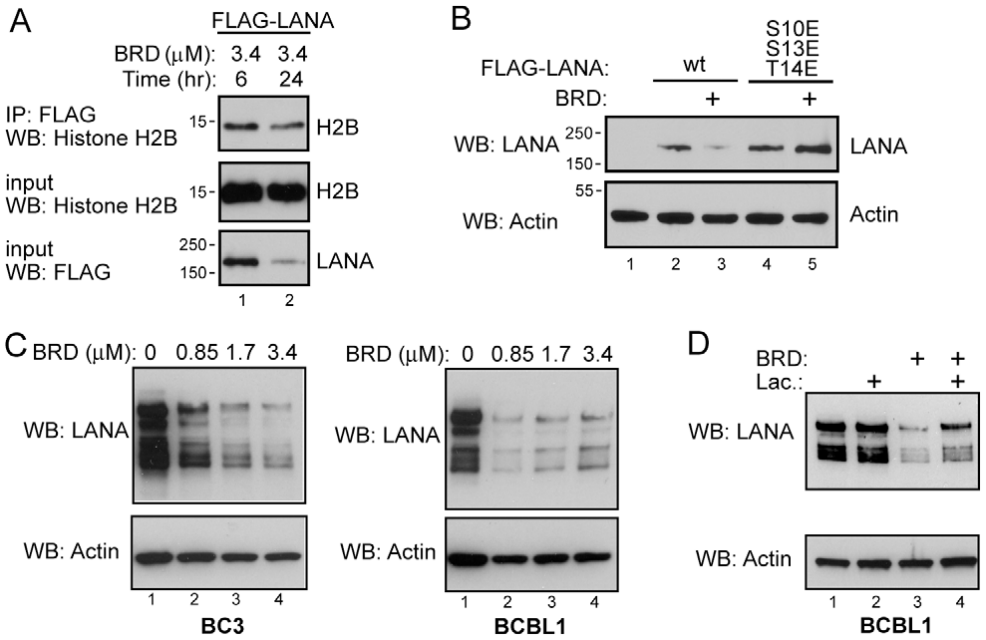
Longer exposure to RSK inhibitor decreases LANA protein levels. A. Western blot of Flag-LANA transfected cells showing that the further reduction in LANA interaction with endogenous histone H2B that occurs upon long-term inhibitor exposure is co-incident with a decrease in Flag-LANA protein levels. B. Western blot comparing the effect of 24 hr exposure to RSK inhibition on LANA levels in wt Flag-LANA or Flag-LANA [S10E, S14E, T14E] transfected cells. C. Western blots showing the effect of increasing doses of BRD 7389 on LANA protein levels in PEL cells. D. Western blot showing the effect of the proteasome inhibitor lactacystin on LANA protein levels in BCBL1 cells treated with RSK inhibitor.

**Figure 8:**
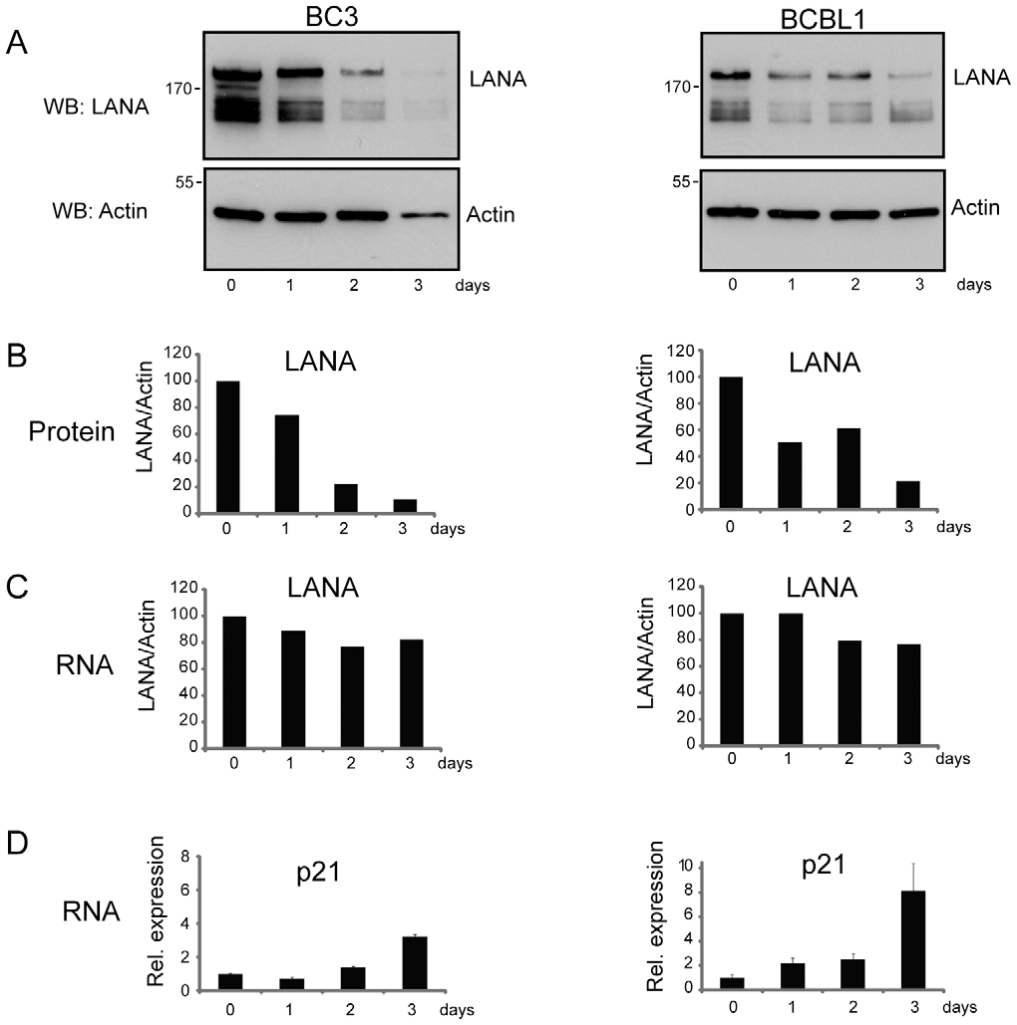
LANA protein loss is post-transcriptional and leads to p21 induction. Comparison of LANA protein (A,B) and mRNA levels (C) with p21 mRNA induction (D) in BC3 and BCBL1 PEL cells treated for 1, 2 or 3 days with 1.7 µM BRD 7389. Protein levels were quantified using Image J and RNA was quantified using real time RT-PCR.

The effect of a longer exposure to RSK inhibitor was examined firstly in transfected 293 cells. Interestingly, when the cells were treated with 3.4 µM BRD 7389 for 24 hr versus 6 hr, there was not only a further decrease in LANA binding to histone H2B but also a decrease in LANA protein levels (Figure 7A). A comparison of LANA protein levels in cells transfected with wt Flag-LANA or the phosphomimetic Flag-LANA [S10E, S13E, T14E] and treated with RSK inhibitor for 24 hr revealed that the decrease in LANA protein levels that occurred with wt LANA was not seen with the triple phosphomimetic mutant (Figure 7B). This result links LANA stability to phosphorylation of the S10, S13 and T14 residues in the chromatin binding domain and to chromatin binding. Exposure of BC3 and BCBL1 PEL cells to 0.85, 1.7 and 3.4 µM concentrations of BRD 7389 for 48 hr resulted in decreased LANA protein levels in each case (Figure 7C). To determine whether the observed loss of LANA was mediated at the level of protein turnover, LANA protein levels were examined in BCBL1 cells treated with 1.7 µM BRD 7389 for 24 hr with or without the addition of the proteosome inhibitor lactacystin for 6 hr prior to harvesting. LANA levels decreased with BRD 7389 treatment as expected, and were partially restored by proteosome inhibition (Figure 7D, lanes 3 and 4). To strengthen the conclusion that the loss of LANA was mediated at the post-transcriptional level, rather than via transcription, BC3 and BCBL1 PEL cells were treated with 0.85 µM BRD 7389 for 1, 2 or 3 days and harvested cells were examined for LANA protein levels by western blotting (Figures 8A and 8B) and for LANA transcription using RT-PCR (Figure 8C). BC3 and BCBL1 cells differed in the kinetics of LANA protein loss over the 3 day time course. However, at each time point there was a greater decrease in the LANA∶Actin protein ratio than in the LANA∶Actin transcript ratio which remained between 76–100% of that in untreated cells. The data are compatible with RSK inhibition affecting LANA protein stability or turnover.

Loss of LANA would be expected to impact on LANA regulated events. LANA is known to bind to p53 and to block p53 mediated responses and release of this inhibition leads to induction of p53 target genes [58]. p53 transcriptionally activates p21, a cyclin-CDK inhibitor that regulates the G1 phase of cell cycle progression. RT-PCR analysis of p21 mRNA levels was performed on the same samples of BC3 and BCBL1 used to measure LANA expression (Figure 8D). Just as the kinetics of LANA protein loss differed between BC3 and BCBL1 cells, the kinetics of p21 induction also differed. The greatest induction of p21 transcripts occurred at 3 days post treatment, a time when LANA protein levels were lowest. Thus loss of LANA protein can be correlated with a downstream change in a LANA mediated function.

### Effect of RSK inhibition on PEL cell growth

**Figure 9:**
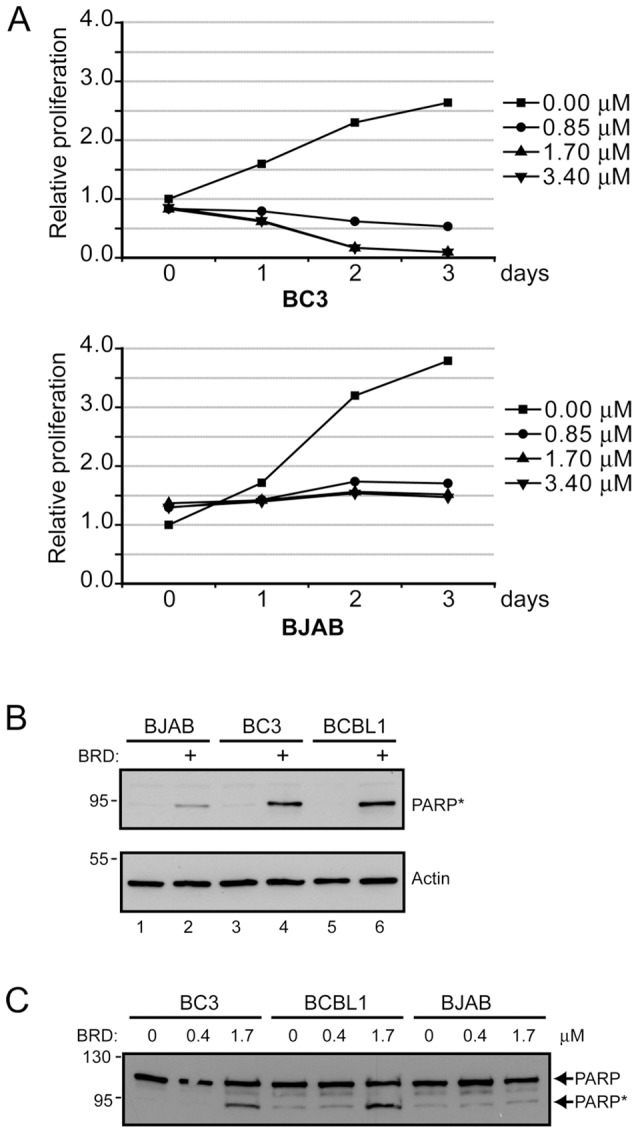
Treatment of PEL cells with BRD 7389 decreases cell proliferation and increases PARP cleavage. A. Growth of BC3 PEL and BJAB B cell lines after treatment with the indicated doses of RSK inhibitor. B. Western blot comparing the levels of cleaved PARP in BJAB B cells and BC3 and BCBL1 PEL cells after treatment for 1 day with 0.85 µM RSK inhibitor. C. Western blot comparing the levels of cleaved (*) and uncleaved PARP in BJAB, BC3 and BCBL1 cells after treatment with 0.4 or 1.7 µM RSK inhibitor.

To determine the effect of the loss of LANA protein on PEL cell growth, duplicate cultures of BC3 PEL cells and KSHV negative BJAB cells were treated with RSK inhibitor and each sample was measured in duplicate for cell viability using the CellTiter-Glo assay. RSK inhibition has been reported to reversibly inhibit proliferation of tumor derived cell lines and indeed all three inhibitor concentrations stopped the growth of both BC3 PEL cells and BJAB B cells (Figure 9A). While, the BJAB cultures maintained their original cell numbers, the BC3 cultures showed a decrease in viable cell numbers over time suggesting that drug treatment was both cytostatic and cytotoxic for BC3 cells. The IC50 for BRD 7389 was 0.27 µM for BC3 PEL cells and 1.88 µM for BJAB B cells.

To further evaluate the difference in loss of viability between inhibitor treated PEL cells and BJAB B cells, BC3, BCBL1 and BJAB cells were treated with 0.85 µM BRD 7389 for 1 day and a western blot was performed to detect PARP cleavage as a measure of caspase induction and apoptosis. The antibody used is specific for cleaved PARP. BRD 7389 treatment induced PARP cleavage that was substantially greater in the PEL cells than in BJAB cells (Figure 9B). The assay was also repeated on cells treated with 0.4 and 1.7 µM BRD 7389 for 1 day and in this case the western blot was probed with antibody that detects both cleaved and uncleaved forms of PARP (Figure 9C). Again PARP cleavage was readily detectable in BCBL1 and BC3 cells and was minimal in BJAB cells.

## Discussion

Protein microarrays and high throughput techniques for kinase expression and purification have previously been used to characterize the yeast kinome and analyze yeast phosphorylation networks [59], [60]. We now describe the use of these technologies to examine phosphorylation of the KSHV LANA protein by human kinases. The ability to screen LANA with 268 different human kinases provided a unique opportunity to examine the potential role of phosphorylation of the chromatin binding domain on LANA function and to identify those kinases able to perform this function.

In vitro kinase screens have limitations. Not all of the kinases that phosphorylate the substrate in vitro will have the appropriate intracellular localization, expression pattern or activating signals to perform this function in the cellular environment while kinases that require specialized reaction conditions or partners such as priming kinases for maximum activity will tend to be under represented. It should be noted that substrates purified from yeast exhibit a limited degree of phosphorylation that can partially support the priming activity required by kinases such as GSK-3. To ensure that we focused on the most biologically relevant LANA partners, kinases that phosphorylated LANA in the initial microarray screen were subjected to database searches for their intracellular localization and only those with a known or predicted nuclear activity were included in subsequent assays. An interesting observation from the initial microarray assay was that the number of kinases phosphorylating LANA and its functional homolog in latency viral DNA replication in EBV, the EBNA1 protein, was an order of magnitude greater than the number detected for most other EBV proteins on the array. This suggests that LANA and EBNA1 functions may be particularly sensitive to regulation by phosphorylation. The extensive overlap in phosphorylating kinases for these two proteins also suggests that they may be subject to regulation by common signaling pathways.

Within the LANA chromatin binding domain, three of the amino acids that had been implicated as critical for histone and chromatin binding in mutagenesis studies were S10, S13 and T14. We tested S13 as a single mutation, S13A, and did not find any impact on LANA binding to histones. This placed the emphasis on S10 and T14 as residues potentially subject to post-translational modification. The ability of phosphomimetic S10E and T14E mutations to restore LANA binding to histone H2B is suggestive of a role for phosphorylation in the regulation of LANA chromatin binding function. Two of the kinases that we identified as phosphorylating these residues, PIM and RSK have been implicated in KSHV lytic replication. Overexpression of PIM1 and PIM3 led to reactivation of KSHV from latency and the role of these kinases was linked to phosphorylation of LANA at serines 205 and 206, an event that abolished LANA mediated repression of RTA [53]. Treatment of PEL cells with the RSK inhibitor BI-D1870 or treatment with inhibitors of ERK signaling also inhibited KSHV lytic replication [61], [62]. The latency and lytic cycles of herpesviruses are usually thought to have opposing requirements. Proteins or pathways that favor latency generally have an inhibitory effect on lytic replication and vice versa. However, a requirement for the activity of certain kinases in both aspects of the KSHV life-cycle need not be contradictory. For example, ERK signaling is also known to be required for establishment of a KSHV infection [63], [64]. ERK signaling, which activates RSK, can have directly opposing effects depending on the duration and strength of the signal and cross-talk with other signaling pathways [65].

We saw no effect on LANA interaction with histone H2B after a short treatment with CK1, PIM1 or GSK-3 inhibitors. However, a short treatment with the RSK inhibitor BRD 7389 decreased LANA binding to histone H2B. All of the RSK family members, RSK1, 2, 3 and 4 are inhibited by BRD 7389. Interestingly, a longer BRD 7389 treatment resulted in a decrease in LANA protein levels in LANA transfected cells and in PEL cells. Treatment of cancer cell lines with RSK inhibitors results in growth inhibition [66], [67]. We observed this same response upon treatment of the BJAB B cell lymphoma cell line and PEL cells. The treated BC3 PEL cells not only stopped growing but also decreased in viable cell numbers suggesting that inhibition of RSK was having an additional impact on these cells. PEL cells are generally wild-type for p53 (BCBL1 cells are heterozygous [11]) and BJAB B cells are p53 mutant. One of the functions of LANA is to block p53 mediated apoptosis through interaction with the p53-MdM2 complex [10], [68]–[70] and through inactivation of GSK-3 [13], [58], [71] and interaction with angiogenin [72]. In RSK inhibitor treated PEL cells, p21 induction by activated p53 occurred later in the time course than induction of caspase cleavage suggesting that the loss of viability of treated PEL cells likely occurred by a p53 independent mechanism.

Overall, our data provide evidence that phosphorylation affects LANA interaction with chromatin and that inhibition of the RSK kinases that phosphorylate the chromatin binding domain has an additional effect on LANA protein levels. The dual outcomes of reduced histone binding and loss of LANA protein stability imply that chromatin binding protects LANA from degradation and contributes to the long half-life demonstrated by LANA in latently infected cells. ERK signaling positively regulates transcription of LANA upon KSHV infection of endothelial cells [73] but the loss of LANA in inhibitor treated cells was mediated at the level of protein turnover rather than transcriptionally. The Ras/Raf/MEK/ERK signaling pathway that leads to RSK activation regulates cell growth, proliferation and survival. The sensitivity of PEL cells to treatment with inhibitors of this pathway may represent a vulnerability that can be exploited to limit the growth of latently KSHV infected cells.

## Materials and Methods

### Protein purification

EBV proteins were purified as 6x-His-GST fusion proteins from yeast using a high-throughput protein purification protocol described previously [74]. Briefly, cultures of 65 yeast strains that express EBV proteins as 6xHis-GST fusions plus yeast expressing 6xHis-GST-LANA (1–329) and 6xHis-GST-LANA (891–1129) proteins were grown, harvested, lysed, and the fusion proteins purified using glutathione beads. After extensive washes, the captured GST-fusion proteins were eluted in elution buffer (50 mM HEPES [pH 7.4] containing 100 mM NaCl, 40 mM reduced glutathione, 0.03% Triton X-100, and 30% glycerol). 6xHis-N-LANA-Avi-tag protein and V5-6xHis EBNA1 (392–641) were purified using the PrepEase His Tagged Protein Purification Kit (USB). The eluate was collected through a filter unit (NUNC) and stored in 384-well plates. Protein products that were successfully purified based on immunoblot analysis were then spotted in duplicate onto microscope slides (Full Moon Biosystems) using a 48-pin contact printer (Bio-Rad). The quality and quantity of the immobilized proteins on these chips was monitored using anti-GST antibody followed by Cy-5 labeled secondary antibody.

### Human kinase purification and activity

Human kinases from 268 recombinant yeast strains were purified as 6xHis-GST-fusion proteins using a previously described high-throughput protein purification protocol [74], [75]. The captured GST-fusion proteins were eluted in elution buffer (50 mM HEPES [pH 7.4] containing 100 mM NaCl, 40 mM reduced glutathione, 0.03% Triton X-100, and 30% glycerol). The eluate was collected through a filter unit (NUNC) and stored at −80°C. Successful purification of human kinases free of contaminating kinase activity from yeast was determined by immunoblot, silver staining and autoradiographic analyses [Newman et al, submitted for publication]. To evaluate the enzymatic activity of each sample, auto- and trans-phosphorylation reactions were performed using a standard liquid-kinase assay with [γ^32^P]-ATP and a mixture of histone H3, myelin basic protein (MBP), and casein.

### Microarray phosphorylation assays

To identify potential LANA phosphorylating kinases, a protocol similar to that described by Zhu et al. was used [56]. Briefly, the protein microarrays were blocked in 1× TBST with 1% bovine serum albumen (BSA) for 1 hr at room temperature with gentle shaking. The arrays were then incubated with individual kinases in reaction buffer (25 mM Hepes at pH 7.5 with 100 mM NaCl, 50 mM Tris-HCl pH 7.5, 55 nM [γ^32^P]-ATP, 1 mM DTT, 10 mM MgCl_2_, 1 mM MnCl_2_, 1 mM EGTA, 1 mM NaVO_4_, 1 mM NaF, and 0.1% NP-40) for 30 minutes at 30°C. Control slides were incubated with kinase reaction mixture without kinase and processed in parallel. The arrays were subjected to three ten minute washes in, firstly, TBS, 0.1% Tween-20 (TBST) and secondly in 0.5% SDS. Arrays were then rinsed briefly with double-distilled H_2_O and dried by centrifugation. Each slide was then exposed to BioMax high resolution X-ray film (Kodak) for 16 hours. Finally, developed films were scanned using an office scanner at a resolution of 4,800 dpi. Captured images from the autoradiographic films were processed using Photoshop. The substrate profile for each image was acquired using GenePix software (Axon). Each protein was printed in duplicate and a protein was scored as a positive hit only if both spots of the same protein showed signal intensity higher than the cutoff value of three standard deviations above the mean. Negative control experiments identified proteins that underwent autophosphorylation on the protein microarrays.

### Plasmids

The Flag-LANA plasmid pMF24 that is deleted for the central repeat region was cleaved with XmaI and AscI and repaired with oligonucleotide insertions to introduce G5A, M6A, R7A, L8A, R9A, S10A, G11A, R12A, S13A or T14A mutations (pMS30 to pMS39 respectively). Glutamic acid substitutions were introduced by site-directed mutagenesis on plasmid DY52 (FLAG-LANA) to generate S10E (pGL621), S13E, T14E (pGL628), and S10E, S13E, T14E (pGL629). These constructions were then used as PCR templates to generate GST-LANA (aa1–329) wt, S10A, S13A and T14A plasmids (pGL485 to pGL488 respectively) and GST-LANA (aa1–50) wt, S10A, S13A and T14A plasmids (pGL502 to pGL505 respectively). The GST-LANA (aa1–50) triple mutant S10A/S13A/T14A (pGL515) was generated using XmaI and AscI cleavage and repair with an oligonucleotide insertion carrying mutated sequences. Full length 3xFlag-LANA carrying the triple mutations S10A/S13A/T14A (pGL628) and S10E/S13E/T14E (pGL629) were generated by PCR mutagenesis in the vector p3xFlag-CMV (Sigma). Full length Flag-LANA (pDY52) has been described previously [76]. 6xHis-LANA (aa1–329)-Biotin Avi-Tag (pGL370) and 6xHis-LANA(aa936–1162)-Biotin Avi-Tag (pGL371) were generated in the bacterial expression vector PAC4 (GeneCopoeia). 6xHis-GST-EBNA1 (386–641), 6xHis-GST-EBNA1 (1–87) and EBNA1 (392–641-V5-6xHis; pGL451D) have been described [56], 6xHis-GST-LANA (1–329) was generated in the same vector background.

### Liquid kinase assays

LANA amino acid 3–21 polypeptides and variants carrying S/T to A mutations were purchased from Peptide 2.0. The purified GST-LANA fusion proteins (0.1 µg) or synthetic polypeptides (250 µM) were incubated with kinase in 25 µl kinase buffer containing 33.3 nM [γ^32^P]-ATP for 30 min at 30°C. Reactions were terminated by heating the mixture at 90°C for 5 min, and the proteins were separated on NuPAGE gels (4 to 12% Bis-Tris; Invitrogen or 4 to 20% Tris-HCl; Biorad). The gels were dried and exposed to MP Hyperfilm (GE Healthcare). For characterization of GSK-3β phosphorylation, peptide substrates were incubated for 30 min at 30°C in (i) kinase buffer with 10 µCi of [γ^32^P]-ATP, (ii) kinase buffer with 10 µCi of [γ^32^P]-ATP and Pim1 (Upstate, #14-573), (iii) kinase buffer with 10 µCi of [γ^32^P]-ATP and GSK-3β (Millipore) and for primed phosphorylations (iv) kinase buffer with 20 µM cold ATP and Pim1 (Upstate, #14-573) followed by incubation with Pim1 inhibitor SMI-4a (0.1 µM; Enzo Life Sciences) for 30 min at 30°C (Millipore). Samples were then reincubated in kinase buffer with 10 µCi of [γ^32^P]-ATP or reincubated for 30 min at 30°C in (v) kinase buffer with 10 µCi of [γ^32^P]-ATP and GSK-3β. Samples were separated by SDS-PAGE (16.5% Tris-Tricine; BioRad) and analyzed by autoradiography.

### Immunoprecipitation and Western blotting

HEK 293T cells grown in 10-cm dishes were transfected with 10 ug of total DNA using calcium phosphate precipitation. 48 h after transfection, cells were lysed in 1 ml of lysis buffer (50 mM Tris (pH 7.9), 100 mM NaCl, 0.5 mM EDTA, 2% glycerol, 0.2% NP-40), plus protease inhibitors (0.5 mM PMSF, 2 ug/ml Aprotinin, and 1 ug/ul leupeptin) and phosphatase inhibitors (Phosphatase Inhibitor Cocktail 1&2; Sigma), sonicated for 10 s, and cleared by centrifugation. Extracts were precleared using protein A/G PLUS-agarose (Santa Cruz Biotechnology, Inc.) and immunoprecipitated with anti-FLAG M2-agarose (Sigma). Beads were washed six times with precipitation buffer and bound H2B was detected by western blotting using rabbit anti-H2B antibody (Abcam). Flag-LANA was detected using rabbit anti-Flag antibody (Sigma) or rat anti-LANA antibody (ABI Advanced Biotechnologies). The effect of kinase inhibition on H2B interaction was examined using the inhibitors CKI-7 (Sigma Aldrich), LiCl (J.T. Baker), SMI-4a (Enzo Life Sciences) and BRD 7389 (Tocris Bioscience). Cells were treated with the proteasome inhibitor lactacystin (Peptide Institute Inc) for 6 hr at a final concentration of 1 µM. PARP cleavage was detected by western blotting using cleaved PARP (ASP214) specific and total PARP antibodies (Cell Signaling). LANA and Actin were detected by rat anti-LANA (Advanced Biotechnologies Inc) and mouse anti-Actin (Sigma).

### Cell growth

BC3 and BCBL1 PEL cells and BJAB B cells were cultured in RPMI 1640 plus 15% fetal bovine serum in 5% CO_2_ at 37°C. Cell growth in cultures treated with BRD 7389 was measured using the Cell Titer-Glo luminescence assay kit (Promega) and luciferase activity was quantified using a Glomax Multi Detection System (Promega).

## Supporting Information

Table S1 Supporting Table S1 lists the kinases that phosphorylated KSHV LANA (N+C) and EBV EBNA1 (N+C) on the protein microarray.
(DOC)
