# Supplementary material for: Phosphorylation of the Chromatin Binding Domain of KSHV LANA
Source: PLoS Pathog. 2012 Oct 18;8(10):e1002972. doi: 10.1371/journal.ppat.1002972 (PMC3475679; doi:10.1371/journal.ppat.1002972)
Supplement: Table S1 — Supporting Table S1 lists the kinases that phosphorylated KSHV LANA (N+C) and EBV EBNA1 (N+C) on the protein microarray. (DOC) [file ppat.1002972.s001.doc]

| **Table S1. Nuclear kinases that phosphorylate KSHV LANA (N+C)** | | | |
| --- | --- | --- | --- |
|  | | | |
| ADRBK2 | GRK5 | MET | PRKX |
| AURKB | GSK3B | MKNK2 | PRPF4B |
| BTK | HIPK1 | MYLK2 | PTK2 |
| BUB1 | HIPK4 | MYO3A | PTK6 |
| CAMK1D | IKBKB | NEK10 | RPS6KA2 |
| CAMK2D | IRAK1 | NEK3 | RPS6KA3 |
| CAMK4 | IRAK3 | NEK6 | RPS6KA4 |
| CAMKK2 | JAK2 | NPR2 | RPS6KA5 |
| CAMKV | KIT | NRBP1 | RPS6KB1 |
| CDC2 | LATS1 | NUAK1 | SCYL2 |
| CDK5 | MAK | NUAK2 | SCYL3 |
| CDKL5 | MAP2K1 | PAK4 | SGK2** |
| CHEK2 | MAP2K3** | PAK6 | SRPK1 |
| CSNK1A1L | MAP2K7 | PBK | SRPK2 |
| CSNK1D | MAP3K11 | PDGFRB | STK17A |
| CSNK1G2 | MAP3K13 | PDPK1 | STK31 |
| CSNK2A1 | MAP3K7 | PIM1 | STK40 |
| CSNK2A2 | MAPK13 | PKN1 | TTK |
| DDR1 | MAPK14 | PKN3 | TYRO3 |
| DMPK | MAPK15 | PLK2 | VRK3 |
| DYRK1B | MAPK6 | PLK4 | WEE1 |
| DYRK2 | MAPK8 | PRKAA2 | WNK1 |
| EIF2AK1 | MAPKAPK5 | PRKACB | ZAP70 |
| FASTK | MARK2 | PRKCB1 |  |
| FGFR1 | MAST1 | PRKCD |  |
| FGFR4 | MAST2 | PRKCZ |  |
|  | | | |
| All kinases except those indicated by ** also phosphorylate EBV EBNA1 (N+C) | | | |
